# Supplementary material for: Bronchoalveolar Lavage Cell Count and Lymphocytosis Are the Important Discriminators between Fibrotic Hypersensitivity Pneumonitis and Idiopathic Pulmonary Fibrosis
Source: Diagnostics (Basel). 2023 Mar 1;13(5):935. doi: 10.3390/diagnostics13050935 (PMC10000588; doi:10.3390/diagnostics13050935)
Supplement: Supplementary file 1 [file diagnostics-13-00935-s001.zip › diagnostics-2217630-supplementary.pdf]

Table S1. Clinical predictors of fibrotic hypersensitivity pneumonitis vs. idiopathic pulmonary fibrosis diagnosis (multivariable logistic regression analysis # further adjusted for estimated pack-years).

Characteristics	OR	95% CI	P-value
**Male**	1.11	0.42 – 2.96	0.828
**Age at diagnosis**	0.91	0.86 – 0.95	<0.001
**Ever smoker**	0.28	0.08 – 0.99	0.048
**Identified exposure**	18.22	5.97 – 55.63	<0.001
**FVC, % predicted**	0.99	0.97 – 1.02	0.491
**FEV_1, %predicted**	0.97	0.94 – 0.99	0.020
**FEV_1/FVC**	0.97	0.93 – 1.02	0.235
**TLC, %predicted**	0.99	0.96 – 1.02	0.562
**TLco, %predicted**	0.97	0.95 – 1.00	0.077
**6MWD, m**	1.00	1.00 – 1.01	0.628
**Desaturation during 6MWT, %**	1.04	0.97 – 1.12	0.254
**Total cell count in BALF**	1.04	1.00 – 1.08	0.036
**Neutrophils in BALF**	1.03	0.97 – 1.10	0.329
**Eosinophils in BALF**	0.99	0.89 – 1.11	0.875
**Lymphocytes in BALF**	1.15	1.08 – 1.22	<0.001
**Lymphocytosis in BALF >20%**	25.25	7.30 – 87.28	<0.001

Adjusted for a priori age, sex and smoking history

BALF: bronchoalveolar lavage fluid; FVC: forced vital capacity, FEV_1: forced expiratory volume in one second, FEV_1/FVC: forced expiratory volume in one second to forced vital capacity ratio; TLC: total lung capacity, TL,co: transfer factor of the lungs for carbon monoxide; 6MWD: 6-min Walk Distance; 6MWT: 6-min Walk Test
